# Rapid Selective Detection of Potentially Infectious Porcine Epidemic Diarrhea Coronavirus Exposed to Heat Treatments Using Viability RT-qPCR

**DOI:** 10.3389/fmicb.2020.01911

**Published:** 2020-08-21

**Authors:** Héctor Puente, Walter Randazzo, Irene Falcó, Ana Carvajal, Gloria Sánchez

**Affiliations:** ^1^ Department of Animal Health, Faculty of Veterinary Medicine, Universidad de León, León, Spain; ^2^ Department of Microbiology and Ecology, University of Valencia, Valencia, Spain; ^3^ Department of Preservation and Food Safety Technologies, Instituto de Agroquímica y Tecnología de Alimentos – Consejo Superior de Investigaciones Científicas (IATA-CSIC), Valencia, Spain

**Keywords:** coronavirus, porcine epidemic diarrhea virus, viability RT-qPCR, infectivity, thermal inactivation

## Abstract

Coronaviruses (CoVs) cause severe respiratory, enteric, and systemic infections in a wide range of hosts, including humans and animals. Porcine epidemic diarrhea virus (PEDV), a member of the *Coronaviridae* family, is the etiological agent of porcine epidemic diarrhea (PED), a highly contagious intestinal disease affecting pigs of all ages. In this study, we optimized a viability real-time reverse transcriptase polymerase chain reaction (RT-qPCR) for the selective detection of infectious and heat-inactivated PEDV. PEMAX™, EMA™, and PMAxx™ photoactivable dyes along with PtCl_4_ and CDDP platinum compounds were screened as viability markers using two RT-qPCR assays: firstly, on PEDV purified RNA, and secondly on infectious and thermally inactivated virus suspensions. Furthermore, PMAxx™ pretreatment matched the thermal inactivation pattern obtained by cell culture better than other viability markers. Finally, we further optimized the pretreatment by coupling viability markers with Triton X-100 in inoculated serum resulting in a better estimation of PEDV infectivity than RT-qPCR alone. Our study has provided a rapid analytical tool based on viability RT-qPCR to infer PEDV infectivity with potential application for feed and feed ingredients monitoring in swine industry. This development would allow for greater accuracy in epidemiological surveys and outbreak investigations.

## Introduction

Coronaviruses (CoVs) cause severe respiratory, enteric, and systemic infections in a wide range of hosts, including human and animals. The emergence of human outbreaks caused by severe acute respiratory syndrome CoV (SARS-CoV) in 2002–2003, by Middle East respiratory syndrome CoV (MERS-CoV) in 2012, and the ongoing pandemic by SARS-CoV-2 has raised the scientific interest in the spillover and severity of zoonoses caused by CoVs.

Porcine epidemic diarrhea virus (PEDV), a member of the *Coronaviridae* family, genus *Alphacoronavirus*, is the etiological agent of porcine epidemic diarrhea (PED), a highly contagious intestinal disease causing a severe diarrhea in pigs of all ages. PEDV was isolated for the first time in 1978 in Europe (Pensaert and de Bouck, 1978) and became a global major concern in swine production after its emergence in North America in 2013, being responsible for worldwide sporadic and large-scale outbreaks (Stevenson et al., 2013; Carvajal et al., 2015; Lee, 2015).

Several studies have suggested the importance of contaminated feed and raw feed materials as a potential source of infection in PEDV transmission (Dee et al., 2016; Trudeau et al., 2016; Schumacher et al., 2018). In fact, PEDV was likely introduced into the United States from China in contaminated swine feed ingredients (Scott et al., 2016), and spray-dried plasma proteins (SDPP), an animal by-product used in diets for weaned piglets, was investigated as a potential source of infection after the first-detected PED outbreaks in Canada (Pasick et al., 2014). Although several studies have confirmed that good manufacturing practices together with at least 2 weeks of storage minimize the risk of infectious PEDV in SDPP (Gerber et al., 2014; Opriessnig et al., 2014; Pujols and Segalés, 2014; Hulst et al., 2019), the detection of PEDV RNA in SDPP still raise concerns in the porcine industry (Foddai et al., 2015).

Nowadays, as is common among viral diseases, PEDV monitoring is usually performed by PCR-based assays detecting viral RNA in collected samples (e.g., stools, environmental samples, feed, and feed ingredients). It is also cost-effective, quick, selective, and quantitatively sensitive. This approach detects the viral nucleic acids of both infectious and noninfectious viruses, finally resulting as being inadequate to report on sample infectivity. Even though cell culture is the gold standard to examine viral infectivity, PEDV detection based on cell culture is hampered by considerable difficulties in achieving isolation of wild-type virus due to multiple factors such as the type of sample, virus titer, cytotoxicity, and genetic aspects, all affecting the final success rate (Oka et al., 2014).

Recently, viability markers, such as monoazide dyes and metal compounds, have been incorporated into qPCR-based methods to predict infectivity of several viruses in different matrices (Fraisse et al., 2018; Randazzo et al., 2018c). This novel assay is of interest for the prevention and control of viral outbreaks with a broad application spectrum, from environmental surveillance (e.g., water) to food and feed safety (Parshionikar et al., 2010; Leifels et al., 2015; Fongaro et al., 2016; Fuster et al., 2016; Prevost et al., 2016; Randazzo et al., 2016, 2018a,b, 2019; Chen et al., 2020; Cuevas-Ferrando et al., 2020). In this study, a viability real-time reverse transcriptase polymerase chain reaction (RT-qPCR) for the selective detection of infectious and heat-treated PEDV has been developed. We screened monoazide dyes and platinum compounds as viability markers using two PEDV RT-qPCR assays. Finally, we optimized a viability RT-qPCR procedure to be applied in porcine serum as a model matrix.

## Materials and Methods

### Viral Strain, Cell Line, and Infectivity Assay

The PEDV strain CV777 provided by Friedrich-Loeffler-Institut (Greifswald, Germany) was propagated and assayed in Vero cells. Vero cells were cultured in Dulbecco’s Modified Eagle’s Medium (DMEM; Biowest, Nuaillé, France) supplemented with 10% heat-inactivated fetal bovine serum (GE Healthcare Bio-Sciences, Austria), 100 units/ml of penicillin, 100 mg/ml of streptomycin, and 0.25 mg/ml of Fungizone® (Antibiotic-Antimycotic 100X, Gibco, Spain). The cells were cultured in T75 flasks at 37°C in a 5% CO_2_ incubator and assayed as complete confluent monolayers in 96-well plates. Ten-fold serial dilutions of PEDV were prepared in DMEM supplemented with 10 μg/μl trypsin (Trypsin 1:250, Gibco) and 100 μl per well were inoculated on a total of eight wells. At 2 h post infection (hpi), 100 μl of post-infection media [DMEM supplemented with 0.3% tryptose phosphate broth (TPB, Sigma, Spain), 100 units/ml of penicillin, 100 mg/ml of streptomycin, 0.25 mg/ml of Fungizone®, and 10 μg/μl trypsin] was added to each well. Plates were incubated at 37°C in a 5% CO_2_ incubator and monitored for cytopathic effects (CPEs) for 3–4 days. PEDV infectivity was calculated by determining the 50% tissue culture infectious dose (TCID_50_) using the Spearman-Karber method after visual inspection of cells for presence of cytopathic effect.

### Extraction, Detection, and Quantification of PEDV

Viral RNA extraction was carried out on 150 μl of viral suspension using the NucleoSpin® RNA virus kit (Macherey-Nagel GmbH & Co., Spain) following the manufacturer’s instructions. Detection of viral RNA was carried out using two assays: (a) EXOone PEDV (EXOPOL, Spain), a commercial kit provided with an internal amplification control (IAC) and amplifying a 191 bp product (referred to as RT-qPCR1), and (b) PrimeScript™ One Step RT-PCR Kit (Takara Bio, USA) using a set of primers and TaqMan probe described by Zhou et al. (2017) targeting at a 140 bp sequence within the highly conserved M gene (referred to as RT-qPCR2). Both RT-qPCR assays were carried out in 96-well plates by using half volumes of all reagents, including RNA template (2.5 μl), in the LightCycler 480 instrument (Roche Diagnostics, Germany). Thermal cycling conditions were as follows: retro-transcription at 45°C for 15 min, initial denaturation at 95°C for 5 min; followed by 45 cycles at 95°C for 15 s, and 60°C for 60 s. RT-qPCR quality controls included negative (nuclease-free water) and positive (RNA) controls added to each PCR plate.

### Intercalating Dye Treatments on Purified PEDV RNA

Photoactivatable dyes such as propidium monoazide (PMAxx™, Biotium, Fremont, USA), ethidium monoazide (EMA™, Geniul, Spain), and PEMAX™ (Geniul, Spain) and metal compounds such as platinum (IV) chloride (PtCl_4_; Acros Organics, Morris Plains, USA) and cis-diamineplatinum(II) dichloride (CDDP; Sigma-Aldrich, St. Louis, USA) were preliminary tested on PEDV purified RNA. Stock solutions were prepared as follow: PMAxx™ and PEMAX™ were diluted in water at 4 mM solution, EMA™ was diluted in dimethylsulfoxide (DMSO) at 2 mM, and PtCl_4_ and CDDP were dissolved in DMSO at 50 mM. All viability markers stock solutions were stored at −20°C for later use.

Purified PEDV RNA extracted with NucleoSpin RNA virus kit was diluted and 150 μl incubated in DNA LoBind tubes (Eppendorf) with photoactivatable dyes and metal viability markers at 20, 50, 500, or 1,000 μM final concentrations. Samples were then incubated with photoactivatable dyes at room temperature (RT) for 10 min in a shaker at 150 rpm and exposed to photoactivation for 15 min using a photo-activation system (Led-Active Blue, GenIUL); alternatively, samples were incubated at RT for 30 min in a shaker at 150 rpm with metal viability markers in DNA LoBind tubes. Each experiment included a purified PEDV RNA sample without viability marker as a positive control. After viability pretreatments, RNA was purified using the NucleoSpin RNA virus and quantified by RT-qPCR as reported above.

### Viability Pretreatments to Discriminate Potentially Infectious and Thermally Inactivated PEDV

Photoactivatable dyes and metal compounds were further tested on PEDV viral particles. PEDV suspensions were prepared in DMEM at concentrations of ~2 and 1 log_10_ TCID_50_/ml and split into two subsamples: a non-treated aliquot (referred to as infectious) and a treated aliquot exposed to 99°C for 5 min (referred to as thermally inactivated). Then, all thermally inactivated subsamples were processed with 100 and 250 μM PMAxx™, 50 and 100 μM PtCl_4_, and 100 and 500 μM CDDP as viability pretreatments before RT-qPCRs. Three types of controls were included in the experiment: infectious PEDV at 2 log_10_ TCID_50_/ml treated with viability pretreatments, and infectious and thermally inactivated PEDV without viability pretreatments. After viability treatment, RNA was extracted and quantified as detailed above.

### Thermal Inactivation Profile of PEDV

In order to further study the thermal inactivation kinetic by using viability markers, PEDV cell culture suspension at ~5 log_10_ TCID_50_/ml were treated at 60, 72, and 95°C for 15 min in a water bath. An aliquot of the viral suspension was kept on ice and used as a control sample. Then, each sample was diluted and split in four subsamples: an aliquot was analyzed with RT-qPCR, two additional aliquots were analyzed with viability RT-qPCR using 100 μM PMAxx™ and 100 μM PtCl_4_ pretreatments, and an aliquot was used to determine the infectivity on Vero cells.

### Performance of Viability RT-qPCR in Serum

Viability RT-qPCR for PEDV was optimized in complex matrix using porcine serum as model. Porcine serum was obtained from a PEDV-free herd. PEDV suspensions at ~2 log_10_ TCID_50_/ml were supplemented with 0.1% Triton X-100 (Fisher Scientific, United States) and incubated with 100 μM PMAxx™, 500 μM PtCl_4_, or 500 μM CDDP in DNA LoBind tubes as detailed previously. Three types of controls were included in the experiments: infectious PEDV treated with viability pretreatments, and infectious and thermally inactivated PEDV without viability pretreatments. After viability treatment, RNA was extracted and detected as detailed above.

### Statistical Analysis

Each experiment was performed in duplicate, and each RNA sample was analyzed in duplicate. Data were statistically analyzed and graphically represented by GraphPad Prism version 8 software (GraphPad Software, USA). Two-way analysis of variance (ANOVA) tested the impact of variables and a multiple comparison procedure (Dunnett’s multiple comparison test) determined significant differences. In all cases, values of *p* < 0.05 were deemed significant.

## Results

### Screening of Viability Markers on PEDV RNA

**Figure 1:**
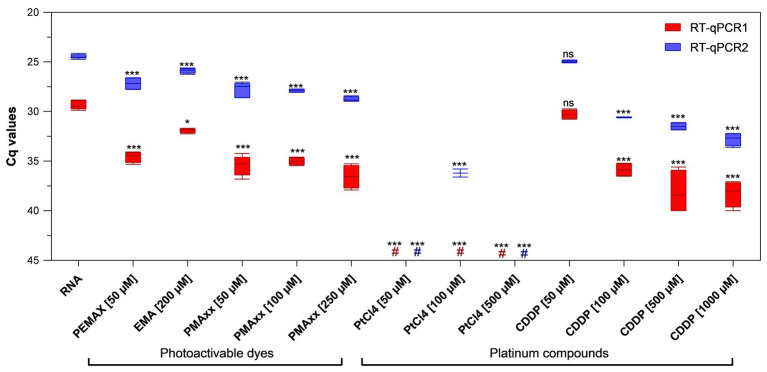
Binding of viability markers to purified porcine epidemic diarrhea virus (PEDV) RNA using two different real-time reverse transcriptase polymerase chain reaction (RT-qPCR) assays. RT-qPCR1 refers to the commercial EXOone PEDV kit; RT-qPCR2 has been described by Zhou et al. (2017). Boxplots show median cycle quantification (Cq) values together with percentiles. Error bars indicate SDs; asterisks indicate significant difference from control: ^*^p < 0.05; ^***^p < 0.001; ns, no significant difference; hash tags (#) represent negatives.

PEMAX™, EMA™, and PMAxx™ photoactivable dyes along with PtCl_4_ and CDDP platinum compounds were initially screened at different concentrations selected from previous studies (Randazzo et al., 2016, 2018c; Fraisse et al., 2018; Chen et al., 2020) on purified PEDV RNA. Among the photoactivable dyes, 250 μM PMAxx™ reduced 7.22 and 4.29 cycle quantification (Cqs) on average with regard to control by using RT-qPCR1 and RT-qPCR2, respectively. In contrast, 50 μM PEMAX reduced 5.23 and 2.70 Cqs, and 200 μM EMA™ reduced 2.56 and 1.40 Cqs by using RT-qPCR1 and RT-qPCR2, respectively (Figure 1). Among the platinum compounds, PtCl_4_ completely removed the amplification signals with both RT-qPCR assays irrespective of the concentration tested, except for residual partial signals with RT-qPCR2 (36.20 ± 0.58 Cq, two out of four replicates). Regarding CDDP, the effect was directly proportional to marker concentrations resulting in 0.92, 6.53, 8.75, and 8.92 Cq reduction for RT-qPCR1, and 0.49, 6.10, 7.02, and 8.31 for RT-qPCR2 (Figure 1). Differences for the IAC were not statistically significant (*p* > 0.05) among the samples (34.41 ± 1.52 Cq on average).

### Performance of Viability Markers on PEDV

**Figure 2:**
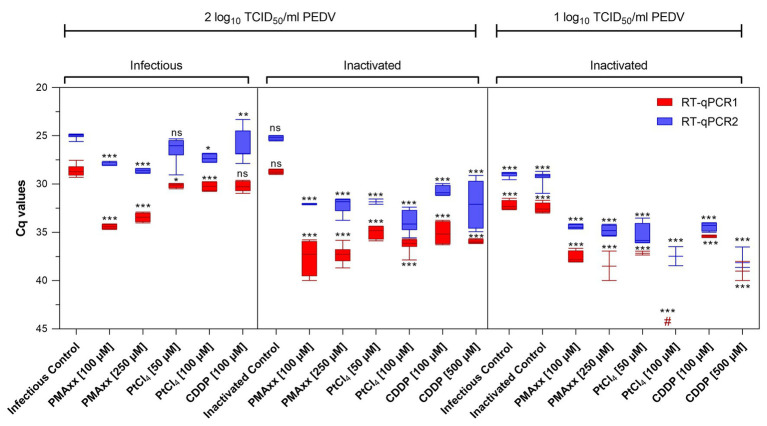
Screening of viability markers on infectious and thermally inactivated (5 min at 99°C) PEDV at two concentrations tested using two different RT-qPCR assays. RT-qPCR1 refers to the commercial EXOone PEDV kit; RT-qPCR2 has been described by Zhou et al. (2017). Boxplots show median Cq values together with percentiles. Error bars indicate SDs; among each group, asterisks indicate significant difference from control: ^*^p < 0.05; ^**^p < 0.01; ^***^p < 0.001; ns, no significant difference; hash tag (#) represent negative.

Infectious and thermally inactivated (99°C for 5 min) PEDV suspensions at ~2 and 1 log_10_ TCID_50_/ml were pretreated with 100 and 250 μM PMAxx™, 50 and 100 μM PtCl_4_, and 100 and 500 μM CDDP before RNA extraction and quantification. No differences between infectious and inactivated controls were detected by either molecular assays (Figure 2).

Pretreatments of infectious virus at ~2 log_10_ TCID_50_/ml with PMAxx™ at 100 and 250 μM reduced 5.78 and 4.81 Cq for RT-qPCR1 and 2.94 and 3.61 Cq for RT-qPCR2 compared to control, while platinum compounds showed minimal reductions (1.57 Cq reduction on average).

On inactivated virus suspensions, PCR signals decreased on average by 8.70 and 6.96 Cq for PMAxx™, by 6.87 and 7.65 Cq for PtCl_4_, and by 6.75 and 6.17 Cq for CDDP, by RT-qPCR1 and RT-qPCR2, respectively.

When PEDV was tested at ~1 log_10_ TCID_50_/ml, PCR signals decreased by 5.57 and 5.29 Cq with PMAxx™, and by 4.76 and 6.75 Cq with CDDP coupled to RT-qPCR1 and RT-qPCR2, respectively. Remarkably, PtCl_4_ completely removed amplification signals with RT-qPCR1 and reduced by 7.07 Cq tested with RT-qPCR2. Differences for the IAC were not statistically significant (*p* > 0.05) among the samples (35.05 ± 1.71 Cq on average).

### Thermal Inactivation Profile of PEDV

The performance of RT-qPCR, PMAxx™-RT-qPCR, and PtCl_4_-RT-qPCR to discriminate between infectious and thermally treated PEDV at 60, 72, and 95°C for 15 min was determined by using RT-qPCR1 and RT-qPCR2 assays and compared to viral infectivity assessed in Vero cells.

**Figure 3:**
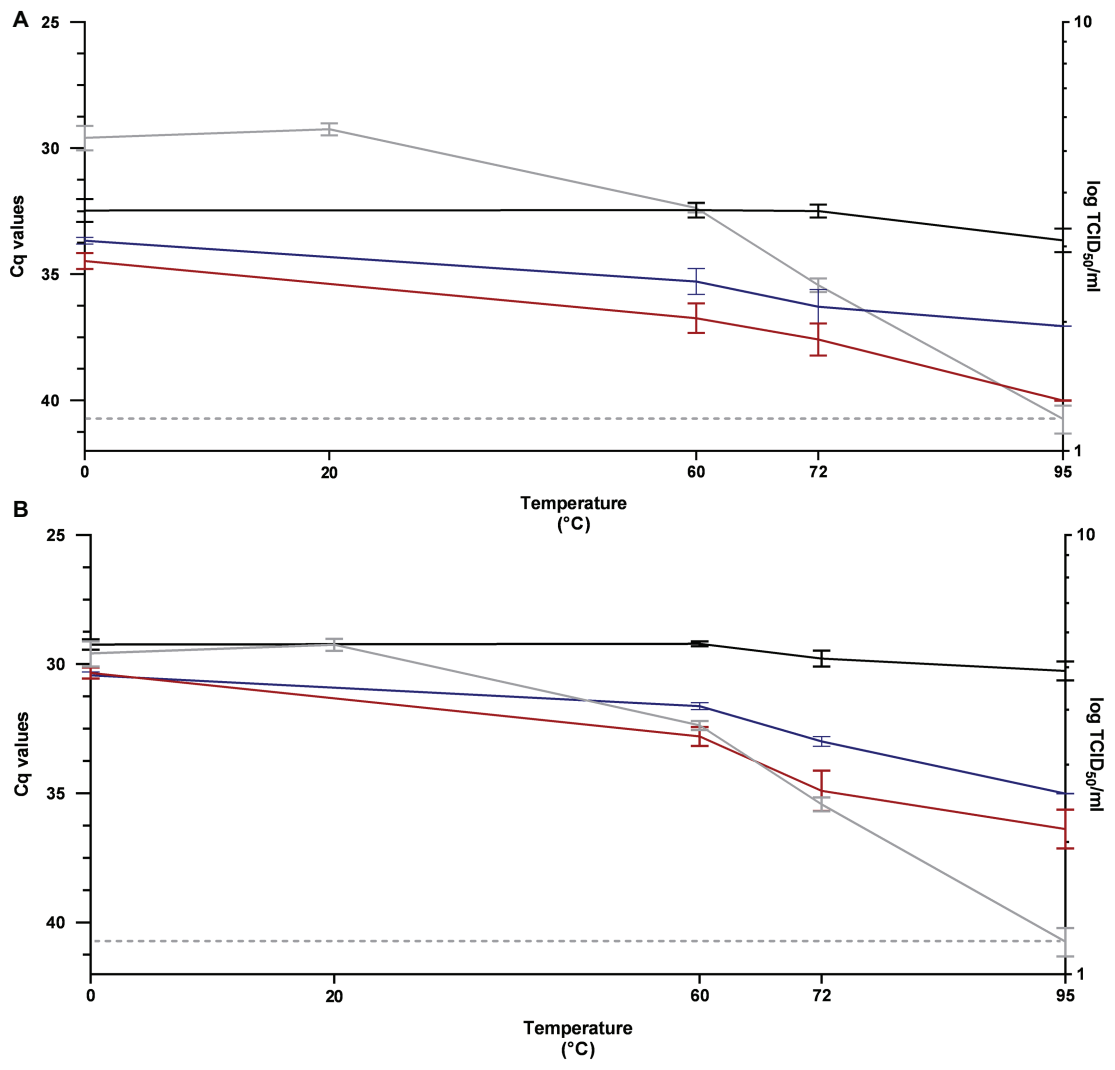
Performance of RT-qPCR (black line), PMAxx™-RT-qPCR (red line), and PtCl_4_-RT-qPCR (blue line) to discriminate between infectious and heat-treated PEDV at 60, 72, and 95°C for 15 min determined by using RT-qPCR1 **(A)** and RT-qPCR2 **(B)** assays and compared to infectivity assayed in Vero cells (gray line). RT-qPCR1 refers to the commercial EXOone PEDV kit; RT-qPCR2 has been described by Zhou et al. (2017).

After pretreatment with 100 μM PMAxx™, signals showed 4.28, 5.09, and 6.35 Cq reductions by using RT-qPCR1 and 3.58, 5.12, and 6.12 Cq reductions by using RT-qPCR2, when heated at 60, 72, and 95°C, respectively (Figure 3).

By using 100 μM PtCl_4_, amplification signals of PEDV heated at 60, 72, and 95°C tested with RT-qPCR1 decreased by 2.83, 3.79, and 3.40 Cq, respectively. The corresponding reductions obtained with PtCl_4_-RT-qPCR2 were 2.41, 3.20, and 4.76 Cq (Figure 3).

Infectivity determined on cell culture showed that PEDV was inactivated by 1.69, 2.94, and >4.19 log_10_ TCID_50_/ml when heated at 60, 72, and 95°C, respectively (Figure 3).

Differences for the IAC were not statistically significant (*p* > 0.05) among the samples (35.20 ± 1.81 Cq on average).

### Optimized PEDV Viability RT-qPCR in Serum as Model Matrix

**Figure 4:**
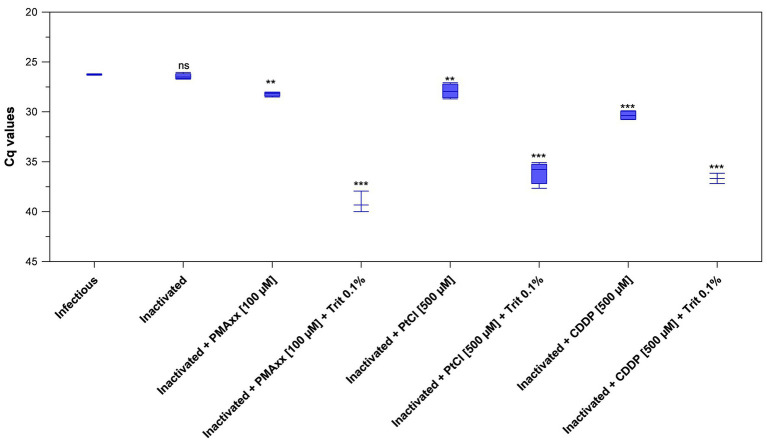
Validation of optimized viability RT-qPCR2 to discriminate between infectious and thermally-inactivated (99°C for 5 min) PEDV in serum. Boxplots show median Cq values together with percentiles. Error bars indicate SDs; asterisks indicate significant difference from control: ^**^p < 0.01; ^***^p < 0.001; ns, no significant difference.

Initially, inactivated PEDV was inoculated in porcine serum and detected by viability RT-qPCR by using 100 μM PMAxx™ and 100 μM PtCl_4_. Results only showed minimal Cq reductions of inactivated PEDV treated with PMAxx™ (+1.41 Cq on average) and PtCl_4_ (−0.64 Cq on average) with respect to inactivated control (32.29 ± 0.40). However, viability RT-qPCR was sharply improved by combining viability markers with Triton X-100 as a surfactant and by increasing PtCl_4_ concentration. By using RT-qPCR2, reductions increased from 1.76 to 12.64 Cq for 100 μM PMAxx™, from 1.48 to 9.63 Cq for 500 μM PtCl_4_, and from 3.91 to 10.23 Cq for 500 μM CDDP with the supplementation of 0.1% Triton X-100 (Figure 4). Given that RT-qPCR2 is not provided with an IAC, 10-fold RNA dilutions were also tested and the results showed no evidence of inhibition.

## Discussion

The urgent need to deeply research into CoV transmission implies the development of analytical tools not only for clinical diagnosis, but also for monitoring additional potential routes of contamination such as animal reservoirs, vectors, the environment, and food and feed. In this scenario, the prompt availability of information on viral infectivity represents the baseline for a rapid and timely public health and veterinary response.

PEDV is a highly contagious enteric swine CoV, which has been associated with devastating outbreaks, particularly in North America and Asia, causing substantial economic losses. The indirect transmission is facilitated by the resistance of the virus in the environment (Carvajal et al., 2015), and fecal contaminated feed and feed ingredients have been pointed as being sources of infection in local and transboundary PEDV outbreaks. Among feed ingredients, pig blood products such as SDPP have been suspected as a potential source of infection (Pasick et al., 2014) and are particularly worrying since possible intrinsic contamination could occur. A research carried out by Canadian authorities showed that the oral inoculation with a PCR positive SDPP with an unknown origin of contamination was capable of reproducing clinical signs of PEDV (Pasick et al., 2014). However, this and other studies have failed to demonstrate infectious PEDV in SDPP supplemented feed (Opriessnig et al., 2014; Pasick et al., 2014).

The combination of temperature and time commonly used during the SDPP manufacturing process together with a storage period of at least 2 weeks at 20°C are the conditions reported to warrant PEDV inactivation (Gerber et al., 2014; Pujols and Segalés, 2014; Hulst et al., 2019). However, the detection of PEDV RNA in feed ingredients using current molecular techniques results in an issue for the swine industry pending to be solved by regulatory policy. In addition, the influence of variations in spray-drying processes has not been sufficiently validated for PEDV [EFSA Panel on Animal Health and Welfare (AHAW), 2014].

The present research shows that viability markers efficiently discriminate infectious from thermally inactivated PEDV in viral suspensions, as well as in serum. Overall, the photoactivatable propidium monoazide dye PMAxx™ pretreatment showed better pattern matching with cell culture than PtCl_4_ RT-qPCR, suggesting that the former is the best approach to infer infectivity of PEDV thermal inactivation kinetics by molecular methods. We further investigated the potential application of viability RT-qPCR in a complex matrix such as the porcine serum. In serum and regardless of concentrations, PMAxx™, and platinum compounds, PtCl_4_ and CDDP, used as pretreatments together with Triton X-100 before RT-qPCR inferred PEDV infectivity better than RT-qPCR alone. Thus, we needed to improve the assay by combining the markers with surfactants as previously reported for enteric viruses such as human and murine norovirus and hepatitis A virus (Coudray-Meunier et al., 2013; Moreno et al., 2015; Randazzo et al., 2016, 2018a).

Our results on thermal inactivation kinetics agree with Zentkovich et al. (2016) who did not recovered viable PEDV after a 10 s or longer treatment with water heated to ≥76°C, even though RNA was detected in all samples regardless of treatment. Similarly, PEDV in feed ingredients was inactivated by 3.9 log_10_ when heated at 90°C for 30 min (Trudeau et al., 2017). As suggested by Weibull kinetic model, 1 log_10_ PEDV reduction could be achieved in swine feed by thermal treatments at 120°C for 16.52 min (Trudeau et al., 2016). However, further experiments are needed to confirm the efficacy of viability RT-qPCR in inoculated feed ingredients exposed to thermal and non-thermal treatments.

Our investigation used two molecular assays since the length of the amplicon and/or the richness of secondary structures of targeted RNA may affect the efficiency of viability RT-qPCR (Contreras et al., 2011; Soejima et al., 2011; Coudray-Meunier et al., 2013; Fraisse et al., 2018; Randazzo et al., 2018c). In our study, viability markers performed similarly, irrespective of RT-qPCR assays. However, the two molecular assays differed in sensitiveness, RT-qPCR2 being the most sensitive. Beside the commercial kit (RT-qPCR1) includes an IAC useful when checking for PCR inhibitors, especially in complex samples, the RT-qPCR2 assay could better fit environmental samples testing with expected low viral concentrations. Moreover, RT-qPCR2 assay targets a highly conserved sequence (membrane gene, M) among PEDV variants (Zhou et al., 2017). Thus, our results could be of valid use to infer the infectivity of wild-type PEDV strains which considerably differ in virulence and genetics and that cannot be routinely isolated in cell culture.

In conclusion, our study provides a rapid analytical tool based on viability RT-qPCR to infer PEDV infectivity with potential application for feed and feed ingredients monitoring in the swine industry, as well as for environmental sampling used for prevention and control programmes. This development would also allow for a greater accuracy in epidemiological surveys and outbreak investigations.

## Data Availability Statement

All datasets presented in this study are included in the article/supplementary material.

## Author Contributions

AC and GS: conceptualization. HP and IF performed the experiments. HP and WR: formal analysis. WR: writing – original draft. AC, GS, IF, HP, and WR: writing – review and editing. All authors contributed to the article and approved the submitted version

### Conflict of Interest

The authors declare that the research was conducted in the absence of any commercial or financial relationships that could be construed as a potential conflict of interest.
